# The Use of Crude Glycerol as a Co-Substrate for Anaerobic Digestion

**DOI:** 10.3390/molecules30173655

**Published:** 2025-09-08

**Authors:** Wirginia Tomczak, Sławomir Żak, Anna Kujawska, Maciej Szwast

**Affiliations:** 1 Faculty of Chemical Technology and Engineering, Bydgoszcz University of Science and Technology, 85-326 Bydgoszcz, Poland; zak@pbs.edu.pl; 2 Faculty of Chemistry, Nicolaus Copernicus University in Toruń, 87-100 Toruń, Poland; akujawska@umk.pl; 3 Faculty of Chemical and Process Engineering, Warsaw University of Technology, 00-645 Warsaw, Poland; maciej.szwast@pw.edu.pl

**Keywords:** anaerobic co-digestion, biodiesel, biogas, crude glycerol, feedstock, inhibition, methane

## Abstract

One of the most interesting applications of crude glycerol (CG) is its use for biogas production via the anaerobic co-digestion (AcoD) process. The main aim of the current study was to provide a comprehensive review on the performance of the AcoD of CG mixed with various substrates. For this purpose, analyses were performed for studies available in the literature wherein one-stage experiments were conducted. To the best of the authors’ knowledge, the present study is the first one which demonstrates an analysis of the main parameters of CG and substrates (e.g., animal manure, sewage sludge, cattle manure and food waste) used for AcoD. Moreover, a detailed analysis of the impact of selected parameters on AcoD performance was carried out. It is demonstrated that the values of key parameters characterizing the CG used for AcoD were within wide ranges. This can be explained by the fact that the composition of CG depends on many factors; for instance, these include the source of oil used for biodiesel production, processing technology, the ratio of reactants, the type of catalyst and the procedure applied. Moreover, performing a literature review allowed us to demonstrate that adding CG to feedstock caused the enhancement of process performance compared to results obtained for mono-digestion. Additionally, it was shown that, in general, increasing the concentration of CG in feedstock led to improvement of the biogas yield; however, a potential inhibitory effect should be considered. Analysis of data available in the literature allowed us to indicate that for most of the experiments performed, a methane (CH_4_) content in biogas higher than 60% was obtained for CG content in feedstock up to 8% *v*/*v*. In addition, it is demonstrated that in order to evaluate the performance of AcoD performed under thermophilic conditions, more studies are required. Finally, it should be pointed out that the present study provides considerable insight into the management of CG.

## 1. Introduction

**Figure 1:**
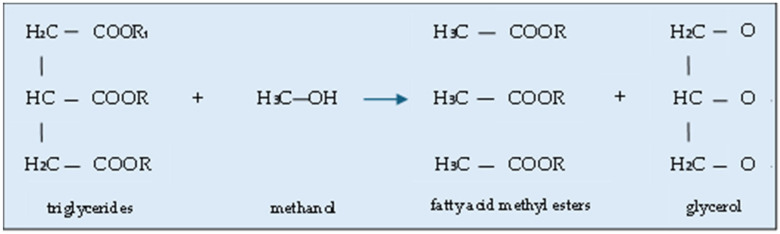
Lipid transesterification. Based on [2,4,5,6,7].

Glycerol (propane-1,2,3-triol, C_3_H_8_O_3_) is a colorless, viscous and nontoxic alcohol with a high boiling point. It has a molecular weight (MW) equal to 92.09 g/mol and a density of 1.261 g/cm^3^. It is a by-product generated during biodiesel production via the transesterification process that uses feedstock such as vegetable oils or animal fat (Figure 1). About one kilogram of crude glycerol (CG) is generated per 10 kg of biodiesel produced [1,2,3,4].

**Figure 2:**
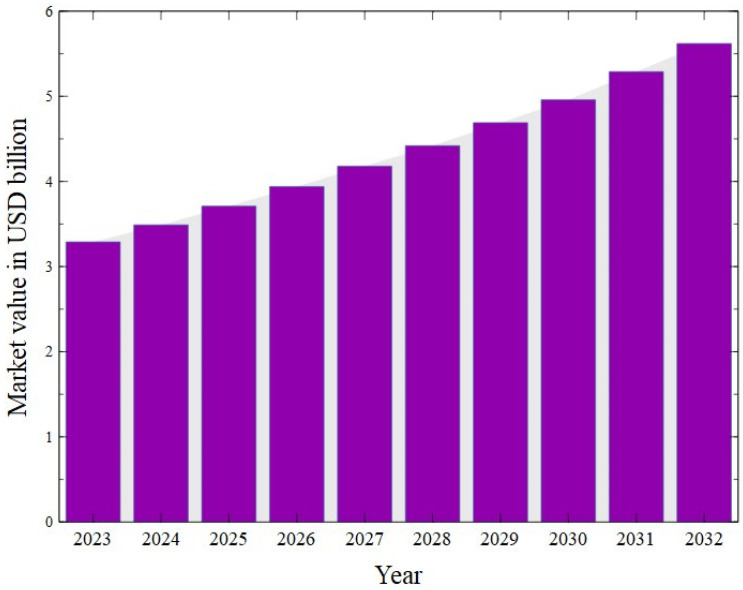
Market value of crude glycerol. Based on [8].

The global market value of CG is growing. Indeed, it was estimated at USD 3.29 billion in 2023 and is expected to reach up to USD 5.6 billion by 2032 [8] (Figure 2). Glycerol has several applications, for instance in the food, cosmetics and pharmaceutical industries [9,10] and polymer technology [7,11]. However, most of them require its purification [12,13,14] since, in general, it contains many impurities and other chemicals that may affect its biological, chemical and physical properties. Among the most common pollutants are methanol, water, heavy metals, salts, soap, free fatty acids, mono-, di- and tri-glycerides as well as methyl esters [15,16,17]. For this reason, untreated CG cannot be discarded into the environment [2]. Importantly, as indicated in a recently published review paper [18], the purification of CG is expensive and often economically unprofitable for both small and medium-sized biodiesel plants. Therefore, it is obvious that the possibilities of using raw glycerol should be expanded.

**Figure 3:**
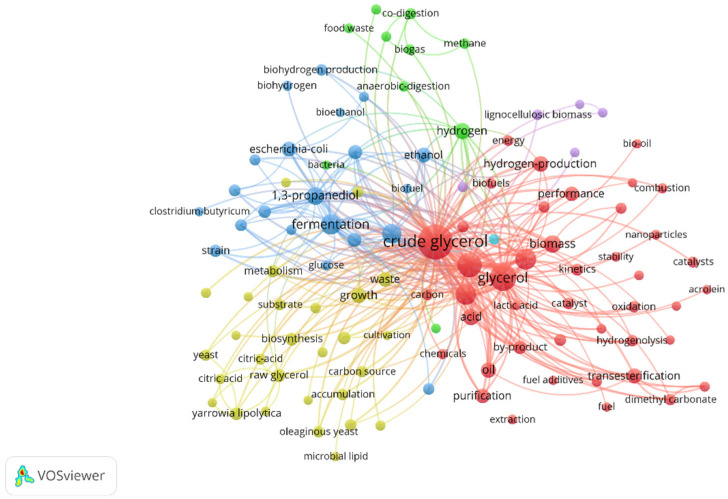
A bibliometric analysis of keywords in papers on crude glycerol published in 2015–2024. VOSViewer software (version 1.6.20).

As recognized in the literature, CG can be used for conversion into value-added products, for instance 1,3-propanediol [19,20,21], 1,2-propanediol [22,23,24], hydrogen [25,26,27] as well as acrylic acid [28,29,30] and lactic acid [31,32,33]. Figure 3 shows the correlation between different keywords used in publications focused on CG (VOSviewer analysis). The map shows five distinct clusters, each ordered by the frequency of terms in the analyzed works. It can be clearly seen that, recently, there has been a particular research focus on the use of CG as a co-substrate for anaerobic digestion (AD) (Figure 3, green cluster). AD, a biological waste management technology [34,35,36,37], is defined as a series of conversion processes of organic compounds to biogas by various synergistically acting facultative or obligatory anaerobic microbial species [38]. In turn, anaerobic co-digestion (AcoD) is the simultaneous anaerobic digestion of a mixture of at least two substrates [39]. It is one of the most economically attractive methods for revalorizing abundant glycerol streams into a biogas that is predominantly methane (CH_4_, 50–75% by volume), carbon dioxide (CO_2_, 25–50% by volume) and minor amounts of other gases, for instance oxygen (O_2_), hydrogen (H_2_), hydrogen sulfide (H_2_S) and water vapor (H_2_O) (g) [38]. It has been widely reported in the literature that the use of CG as a co-substrate for biogas production is attractive due to the improvement in the process yield. This can be explained by the fact that glycerol is a source of additional carbon which can be consumed by specific microorganisms and converted to methane [40]. In addition to increased efficiency leading to improved economic viability of biogas plants, the AcoD process has other advantages compared to mono-digestion, such as the following [41]:

(i) Dilution of toxic substances;
(ii) Increased organic loading rate;
(iii) Nutrient balance;
(iv) Synergistic effects on microorganisms;
(v) Adjustment of the pH and moisture content.

In addition, it offers several environmental benefits, including a reduction in waste disposal and greenhouse gas emission as well as the possibility of nutrient (mainly nitrogen (N) and phosphorus (P)) recovery [42,43,44].

The performance of AcoD of various types of waste has been thoroughly discussed in several recently published review papers [45,46,47,48,49,50,51]. However, to the best of our knowledge, so far, no review article has been published on the use of CG as a co-substrate for AcoD.

Therefore, the main aim of this study was to provide a comprehensive review on the performance of anaerobic co-digestion process of CG mixed with various substrates. For this purpose, an analysis has been performed for studies wherein one-stage experiments have been conducted. It is related to the fact that, as has been thoroughly discussed in our previous paper [52], single-stage AD provides several significant advantages, such as low costs, higher sludge stabilization compared to few-stage systems, as well as low requirements for advanced control and monitoring. To the best of the authors’ knowledge, this paper is the first one to demonstrate a detailed analysis of the reported characteristics of:

(i) Crude glycerol;
(ii) Substrates; used for anaerobic co-digestion, as well as:
(iii) The impact of selected factors (CG content, C/N ratio, temperature and pH) on the AcoD performance with the use of crude glycerol.

Hence, it should be clearly pointed out that our study provides considerable insight into the management of CG by its utilization for biogas production via AcoD.

## 2. Characteristics of Feedstocks

### 2.1. Crude Glycerol

It is well known that crude glycerol is an ideal co-substrate in the AcoD process. Indeed, it is characterized by several advantages, including:

(i) High anaerobic biodegradability [40,53,54,55];
(ii) High organic matter content [56];
(iii) Being a highly concentrated waste [40,54];
(iv) Easily biodegradable character [55];
(v) Ease of long-term storage [53].

**Figure 4:**
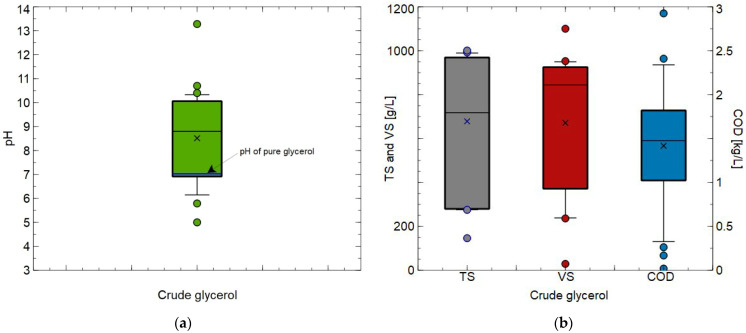
Characteristics of crude glycerol used as a co-substrate for anaerobic co-digestion process reported in the literature: (**a**) pH; (**b**) TS, VS and COD.

Despite this, up to now, the characteristics of CG used as a co-substrate for AcoD have not been dealt with in depth. Therefore, in the present study, the main parameters of CG reported in the literature have been thoroughly investigated (Table 1). It has been found that the glycerol concentration in CG used for AcoD was from 46.5% [17] to 80% [57]. CG characterized by the lowest pH value (5.0 ± 0.1) was obtained from a biodiesel production company that produces biodiesel mainly from the following substrates: soybean oil, sunflower oil as well rapeseed oil [53,58]. In turn, the CG with the highest pH (13.28) came from a biodiesel-making company which transforms used cooking oils into biodiesel fuel [59]. It has been determined that the average pH of CG used for AcoD was equal to 8.51, while the median was 8.80 (Figure 4a). These values are significantly higher than that of pure glycerol, which is equal to around 7. Obviously, the noted difference is related to the fact that, as has been indicated in Section 1, CG contains several various impurities, including for instance soap, glycerides and methyl esters. On the other hand, the reported values of CG density (between 1.052 ± 0.1 kg/L and 1.26 kg/L) were similar to that of pure glycerol (1.261 kg/L) [60].

A closer look at CG characteristics brings to light that both total solids (TS) and volatile solids (VS) values had wide ranges. The average values of the above-mentioned parameters were equal to 678.4 g/L (median: 718.0 g/L) and 671.4 g/L (median: 844.0 g/L), respectively (Figure 4b). The lowest values of TS (146 g/L) and VS (29 g/L) have been reported for CG that came from biodiesel industry, wherein soybean oil and animal fat transesterification is performed [61]. In turn, the highest values of TS (1000.8 g/L) and VS (1100 g/L) were noted for CG collected from industry, wherein biodiesel is generated mainly from animal fat and vegetable soy oil [40,62]. Finally, performing the literature review allowed us to demonstrate the values of chemical oxygen demand (COD) which provides the amount of oxygen required for the organic matter to be oxidized by oxidizing agents [63,64]. In the present study, it has been found that the average and median values of COD for CG used for AcoD were equal to 1.38 kg/L and 1.43 kg/L, respectively (Figure 4b). The lowest and highest values of COD were equal to 0.019 kg/L [65] and 2.925 kg/L [66]. It is worth noting that, in general, the CG used in the analyzed studies was characterized by high content of total nitrogen (TN), from 100 mg/L [67] to 1700 mg/L [68].

To sum up, it should be pointed out that the values of key parameters characterizing the CG used for the AcoD were within wide ranges. It can be explained by the fact that the composition of raw glycerol depends on several factors, such as the source of oil used for the biodiesel production, processing technology as well as ratio of reactants, type and concentration of catalyst and procedure applied [69,70,71].

**Table 1:** Main characteristics of crude glycerol used for anaerobic co-digestion reported in the literature.

Glycerol [g/L] or [%]	pH	TS [g/L] or [%] or [(g/kg)]	VS [g/L] or [%] or [(g/kg)]	Density [kg/L]	Ash [g/kg] or [%] or [(g/L])	COD [kg/L]	TN [mg/L] or [%]	TP [mg/L] or [g/kg]	C [%]	Na [mg/L] or [mg/kg]	Methanol [g/L] or [%]	C/N [–]	Ref.
-	8.39 ± 0.02	488 ± 3.64	488 ± 3.64	-	-	1.83000 ± 0.02121	-	-	-	-	-	-	[3]
46.5	10.4	78.24	95.03	-	-	-	-	-	-	-	5.05	-	[17]
70	6.35 ± 0.09	1000.8	947.8; 94.8	-	4.8	1.023	-	-	-	-	-	-	[40]
-	5.0 ± 0.1	-	-	1.25 ± 0.1	2.8 ± 0.1	-	372 ± 21	9.6 ± 1.3	-	-	-	-	[53]
660 ± 19	-	933 ± 27	844 ± 24	-	-	0.660 ± 0.019	-	-	-	-	1.2 ± 0.1	-	[54]
80	8–9	-	-	-	-	1.140	-	-	-	16,939	-	-	[58]
-	5.0 ± 0.1	-	-	1.25 ± 0.1	2.8 ± 0.1	-	372 ± 21	9.6 ± 1.3	-	-	-	-	[59]
41.67	13.28	80.98	75.23	-	-	-	-	-	-	-	-	-	[61]
-	6.4	146	29	-	-	1.0286	-	-	-	-	-	-	[62]
-	-	-	1100	-	-	1.43	-	-	-	-	-	-	[65]
-	9.7	87.47	-	1.29	-	0.01936	1.47	-	18.88	-	-	12.84	[66]
-	8.86 ± 0.01	279.53 ± 3.34	254.96 ± 2.94	-	-	2.925 ± 0.007	1.76 ± 0.01	-	720.03 ± 0.27	-	-	409 ± 0.03	[67]
-	9.5 ± 0.1	662 ± 28	623 ± 27	-	-	2.409 ± 0.454	100	-	-	-	-	-	[68]
-	8.8	969	910	-	-	1.76	1700	-	-	-	-	949	[72]
-	10.3 ± 0.3	717.99 ± 4.1	649.12 ± 2.1	-	-	1.82312 ± 0.0063	-	-	-	-	-	-	[73]
-	8.8	969	910	-	-	1.76	1700	-	-	-	-	949	[74]
394	8.5	986	940	-	-	1.85	-	-	-	-	-	-	[75]
-	-	80.98	75.23	-	-	-	-	-	-	-	-	-	[76]
-	-	95	-	-	-	1.532	-	-	-	-	-	-	[77]
-	-	81.77	72.93	-	-	-	-	-	-	-	-	-	[78]
-	6.3	-	-	-	-	-	-	-	-	-	-	-	[79]
70	6.35	1000.8	947.8; 94.8	-	-	1.023	-	-	-	-	-	-	[80]
-	9.0 ± 0.1	277 ± 12	240.2 ± 9.5	1.10 ± 0.1	-	1.631 ± 0.025	-	-	-	-	-	-	[80]
-	10.2 ± 0.1	275 ± 12	236.0 ± 9.0	1.10 ± 0.1	-	1.486 ± 0.021	-	-	-	-	-	-	[81]
-	6.83	24.44	54.42	-	-	-	-	-	-	-	-	-	[82]
49.4 ± 0.3	7.6 ± 0.2	-	-	-	4.8 ± 0.5	-	-	-	-	16760 ± 0053	5.6 ± 0.8	-	[83]
-	-	(787.6 ± 8.2)	(761.4 ± 9.2)	-	-	-	<0.06 ± 0.1	-	35.6 ± 0.2	49.3 ± 7.5	-	-	[84]
47 ± 8.6	-	79.5	72.1	1.02	-	1.477 ± 0.235	-	-	-	-	-	-	[57]
74	8.0	-	-	1.26	-	1.119	-	-	-	-	0.019	-	[85]
50.6	10.7	-	-	-	-	1.000	-	-	-	-	7.1	-	[86]
-	10.02 ± 0.01	(62.69 ± 9.00)	(56.61 ± 9.29)	-	6.08 ± 0.33	0.16850 ± 0.00495	<0.01	-	55.90 ± 0.10	-	-	-	[87]
-	8.8	969	910	-	-	1.760	1670	71500	-	-	-	949	[88]
-	7.0	(850.5)	(850.3)	1.3	-	-	-	-	-	-	-	-	[89]
-	10.1 ± 0.1	-	-	1.052 ± 0.1	7.2 ± 0.4	0.262 ± 0.009	-	-	59.4 ± 0.1	-	-	-	[90]
-	10.30 ± 0.10	870.34 ± 0.10	870.10 ± 0.10	-	-	1.9740 ± 0.0031	<0.05	-	88.04 ± 0.05	-	-	-	[91]
-	-	992.2 ± 4.2	952.6 ± 4.5	-	-	-	-	-	-	-	-	-	[92]
75	-	-	-	-	5	-	-	-	-	-	<1	-	[93]

TS—total solids; VS—volatile solids; COD— chemical oxygen demand; TN—total nitrogen; TP—total phosphorus; C—carbon; C/N—carbon to nitrogen ratio; -—not available.

### 2.2. Substrates

It is important to take into consideration the fact that CG is characterized by a lack of nutrients which are essential for the AD process. To solve this problem, it may be co-digested with several various substrates. Performing the literature review allowed us to demonstrate that the most commonly used substrates include animal manure, sewage sludge and domestic sludge, cattle manure, leachate, distillery wastewaters as well as food waste (Table 2).

Animal manure is defined as by-products generated by animals grown to produce for instance meat, milk and eggs. Roughly speaking, it is a source of nutrients for crops and grasslands [94]; however, its composition varies between animal species and manure types [95]. According to the literature, the main limitation of the use of animal manure as a substrate for AD is the low carbon-to-nitrogen (C/N) ratio, which decreases the microorganism activity [89]. Furthermore, mono-digestion of animal manure may be limited by high nitrogen concentration, which may inhibit methanogens [96]. In the present study, it has been determined that the animal manure used as a substrate for AcoD was characterized by TN in the range between 0.84% [65] and 4.08 ± 0.05% [91]. In addition, it has been found that the substrate was characterized by TS and VS from 20.7 ± 0.1 g/L [90] to 255.97 ± 1.2 g/L [72] and from 13.6 ± 0.2 g/L [90] to 211.43 ± 2.7 g/L [72], respectively. Other parameters, such as pH and COD, also varied significantly. Indeed, values of pH were in the range from 6.51 ± 0.54 [72] to 7.7 ± 0.1 [78], while values of COD were between 27.5 ± 0.4 g/L [90] and 171.26 ± 3.4 g/L [72]. The use of animal manure for AcoD with CG has several advantages. For instance, since animal manure is characterized by high alkalinity, it ensures a buffering capacity for the accumulation of volatile fatty acids (VFAs) produced during digestion. In addition, macro- and micro-nutrients occurring in this substrate have the positive effect on the bacterial growth [87]. In recent years, many researchers have presented a review of studies on the anaerobic digestion of animal manure [47,97,98,99,100].

Sewage sludge is the solid component generated during wastewater treatment [101]. It is mainly composed of the solid portion of the sewage and the microbial cells [102]. Moreover, as indicated in [96], it is characterized by low organic loads. The application of sewage sludge as a substrate for the AD process has been highlighted by an important body of research [103,104,105,106]. Results obtained in the current study documented that the values of the main properties of sewage sludge, such as pH, TS, VS, COD and TN, varied significantly. With regards to pH, the reported values were in the range from 5.65 ± 0.11 [107] to 7.1 [86] and the pH average and median were 6.71 and 6.9, respectively. Values of TS (average: 35.1 g/L, median: 38.25 g/L) and VS (average: 20.11 g/L, median 21 g/L) were from 25.6 g/L [85] to 41.1 ± 3.6 g/L [54] and from 19.6 g/L [86] to 34.3 ± 3.1 g/L [54], respectively. In turn, values of COD (average: 36.89 g/L, median: 38.7 g/L) were from 21.98 g/L [77] to 51.9 ± 6.4 [54], while concentration of TN (average: 731.58 mg/L, median: 791 mg/L) was between 15.2 ± 0.1 g/L [40] and 1200 ± 600 [67].

Cattle manure, a by-product of cattle farming, is a rich source of carbon and nitrogen [108]. Unfortunately, the main characteristics of cattle manure used as a co-substrate for biogas production have been presented only in a few studies [76,82,87,109]. In the above-mentioned investigations, the pH values of cattle manure were in the range from 7.16 ± 0.06 [87] to 7.5 ± 0.5 [82]. In turn, the total COD was from 8.000 ± 0.354 g/L [87] to 293 g/L [76]. In the literature, there are available review studies focused on the methods of cattle manure treatment [110,111], control of bacteria in cattle manure [112], as well as the impact of co-substrate on biogas production with the use of cattle manure [113].

Leachate is a by-product derived from municipal solid wastes formed in several places, for instance landfills, composting plants, and transfer stations [114]. In general, its quality depends on many factors, such as the waste composition and biological, chemical, and physical conditions in a landfill body as well as location, landfill time and seasons [114,115]. According to [61], due to high water content, leachate can be a solvent for CG and can provide specific macro- and micronutrients for growth of bacteria during the AcoD process. Sources, nature, composition and treatment methods of landfill leachate have been thoroughly discussed in [116,117,118,119]. Leachate used as a co-substrate for biogas production via AcoD has been described in [61]. It has been characterized by pH of 7.3 as well as of TS, VS, and COD of 10.79 g/L, 5.53 and 4.04 g/L, respectively. Moreover, concentration of phosphorus (TP), alkalinity and ammonium (NH_4_^+^-N) concentration were 18.51 mg/L, 4160 mg/L and 0.67328 g/L, respectively.

Distillery wastewater is the aqueous waste generated during ethanol production. According to [120] it may pose a significant environmental issue since it may contaminate water sources in several ways. In general, it contains various salt and heavy metals and is characterized by high COD [121]. However, its characteristics depend on the feed stock used [122]. As a matter of fact, in the present study, it has been noted that the studies focused on the use of distillery wastewater for AcoD with the CG are very limited [66]. In the above-mentioned study, distillery wastewater was characterized by pH of 3.52 ± 0.02, TS and VS of 44.08 ± 0.60 g/L and 40.67 ± 0.58 g/L, respectively, as well as VFA of 2.21426 ± 0.00001 g/L and COD of 57.00 ± 0.71 g/L. It is worth noting that a detailed overview on the management of distillery wastewater can be found, for example, in [121,123,124].

Food waste refers to uneaten food and material discarded due to color or appearance [125]. According to [126], in the European Union, about 90 million tonnes of food is lost every year. In recent years, it has attracted great attention due to its important resource, environmental and social impacts [127]. Roughly speaking, food waste is a source of fat, starch, protein and cellulose [128]. In the present study, it has been documented that the characteristics of food waste used for AcoD with crude glycerol are very limited. Food waste used as a substrate for biogas production was characterized by TS, VS and VFA equal to 41.90%, 40.43% and 41.25 g/L, respectively [77] (Table 2). The possibilities of food waste management have been discussed in [129,130,131,132]. Moreover, its application in biogas production has been presented in our recently published review paper [52] and others [133,134,135,136,137].

**Table 2:** Main characteristics of substrates used for anaerobic co-digestion with crude glycerol reported in the literature.

Substrate	pH	TS [g/L] or [%] or [(g/kg)]	VS [g/L] or [%] or [(g/kg)]	VFA [g/L]	Total COD [g/L] or [g/kg]	TN [mg/L] or [%]	TP [mg/L] or [%] or [(g/kg)]	C [%]	Alkalinity [mg/L]	NH_4_^+^-N [g/L] or [g/kg]	C/N [–]	Ref.
animal manure	-	64.6	47.5	3.3	43.8	-	-	-	13600	-	-	[62]
animal manure	7.1	25.07	4.76	-	-	0.84	-	15.39	-	-	18.32	[65]
animal manure	6.92 ± 0.2	255.97 ± 1.2	211.43 ± 2.7	-	171.26 ± 3.4	-	-	-	-	-	-	[72]
animal manure	7.7 ± 0.1	30.7 ± 1.5	21.2 ± 1.0	2.0 ± 0.5	33.0 ± 3.2	-	-	-	7400 ± 400	0.46 ± 0.01	-	[78]
animal manure	-	-	-	-	-	2.3 ± 0.1	-	44.9 ± 0.2	-	-	-	[83]
animal manure	7.5	-	-	-	-	-	-	-	-	4.4	16.4	[89]
animal manure	-	20.7 ± 0.1	13.6 ± 0.2	-	27.5 ± 0.4	-	-	-	-	4.4	-	[90]
animal manure	-	46.2 ± 0.2	32.1 ± 0.2	-	58.7 ± 0.4	-	-	-	-	4.4	-	[90]
animal manure	6.51 ± 0.54	199.86 ± 0.10	167.55 ± 0.10	-	137.83 ± 1.34	4.08 ± 0.05	-	49.62 ± 0.05	-	-	-	[91]
animal manure	-	71.0 ± 0.6	48.0 ± 0.8	-	-	-	-	-	-	-	-	[92]
animal manure	7.5 ± 0.1	23.34 ± 0.24	15.49 ± 0.43	-	-	-	-	-	-	-	-	[93]
sewage sludge	6.52 ± 0.09	41.1 ± 1.0	22.3 ± 0.6; 54.2	-	38.7 ± 0.1	15.2 ± 0.1	-	29.8	960	-	17	[40]
sewage sludge	6.8 ± 0.2	35.4 ± 3.1	26.1 ± 2.8	-	35.2 ± 2.4	1042 ± 157	845 ± 58	-	-	-	-	[53]
sewage sludge	6.8 ± 0.5	41.1 ± 3.6	34.3 ± 3.1	0.2 ± 0.1	51.9 ± 6.4	-	-	-	-	0.8 ± 0.3	-	[54]
sewage sludge	7.3	-	-	-	40	1200	300	-	1600	-	-	[67]
sewage sludge	7.1 ± 0.4	26.3 ± 4.5	19.7 ± 5.6	-	38.8 ± 14.2	1200 ± 600	-	-	-	-	-	[77]
sewage sludge	-	2.03	0.83	3.48996	21.98333	-	-	-	-	-	-	[79]
sewage sludge	6.52	41.1 ± 1.0	22.3 ± 0.6; 54.2	-	38.7 ± 0.1	392.3	15.2 ± 0.1	29.8	960	0.0837 ± 0.0004	17	[84]
sewage sludge	-	-	-	-	17.4 ±3.6	540	-	-	-	0.0998	-	[57]
sewage sludge	7.1	4.85	61.69	-	-	-	-	-	-	-	-	[57]
sewage sludge	7.0	25.6	15.8	-	-	5.4	3.4	34.7	-	-	-	[85]
sewage sludge	7.12	-	19.6	-	-	-	-	-	-	-	-	[86]
sewage sludge	5.65 ± 0.11	-	-	-	49.41 ± 5.53	-	-	-	-	-	-	[107]
cattle manure	-	19	83	-	293	-	-	-	-	-	-	[76]
cattle manure	7.5 ± 0.5	-	-	-	-	-	-	-	-	-	-	[82]
cattle manure	7.16 ± 0.06	-	-	1.691 ± 0.012	8.000 ± 0.354	2.75 ± 0.39	-	36.05 ± 1.33	-	0.1661 ± 0.0004	-	[87]
cattle manure	7.4 ± 0.02	(84.10 ± 0.26)	(40.31 ± 0.55)	-	(48.11 ± 0.27)	-	-	-	-	-	-	[109]
leachate	7.3	10.79	5.53	-	4.04	-	18.51	-	4160	0.67328	-	[61]
distillery wastewater	3.52 ± 0.02	44.08 ± 0.60	40.67 ± 0.58	2.21426 ± 0.00001	57.00 ± 0.71	1.88 ± 0.02	-	47.06 ± 0.54	6.67 ± 0.02	-	25.03 ± 0.07	[66]
food waste	-	41.90	40.43	41.26332	-	-	-	-	-	-	-	[77]
dairy wastewater	-	12.50	11	-	-	-	-	-	-	-	-	[75]
dairy wastewater	7.86	0.55	77.84	-	-	-	-	-	-	-	-	[81]
milk wastewater	-	4.42	3.59	21.56523	54.650	-	-	-	-	-	-	[77]
seafood wastewater	6.3	9.37	7.76	2.230	10.4	870	53.6	-	2560	-	11	[88]
agro-industrial waste	4.80 ± 0.20	15.00 ± 2.23	13.00 ± 1.98	-	99.00 ± 8.77	2200 ± 1150	2500 ± 3760	-	-	-	-	[138]
sardine wastewater	6.8	8.5	6.4	-	12.0	1500	-	-	-	-	11	[68]
sardine wastewater	6.8	8.5	6.4	-	14.4	870	-	-	-	-	11	[73]
meat and bone meal	-	98.46	68.47	-	-	10.52	4.07	44.09	-	-	4.19	[59]
meat and bone meal	-	98.46	68.47	-	-	-	-	-	-	-	-	[75]
domestic sewage	7.0 ± 0.3	0.183 ± 0.073	-	-	-	-	-	-	-	-	-	[56]
municipal solid waste	4.3 ± 0.5	40.8 ± 14.4	30.5 ± 10.9	-	27.6 ± 2.9	480 ± 29	64 ± 12	-	-	0.014. ± 0.0025	-	[58]
palm oil mill final	4.7	66.20	44.12	13.70	88.80	-	-	-	940	-	-	[74]

TS—total solids; VS—volatile solids; COD—chemical oxygen demand; TN—total nitrogen; TP—total phosphorus; C—carbon; C/N—carbon to nitrogen ratio; -—not available.

## 3. Performance of Anaerobic Co-Digestion Process

Table 3 shows the performance of the AcoD process with the use of CG as a co-substrate. In the present study, the analysis has been performed for continuous experiments with two and three substrates. The AcoD performance was evaluated in terms of biogas and CH_4_ production rate, biogas and CH_4_ yield and CH_4_ content in biogas.

It can be seen that most of the studies have been performed at a laboratory scale. Indeed, only a few studies [3,57,67,84,86,109,138] provided results obtained for a pilot-scale AcoD. For instance, in [57] the AcoD process of CG and sewage sludge has been investigated with the use of a pilot system consisting of a single-stage 50 L continuously stirred tank reactor (CSTR). In turn, in [138] biogas production was conducted via AcoD of CG and agro-industrial waste in a 300 L pilot plant. To sum up, it can be indicated that further studies with the use of a pilot-scale installation are required. Ormaechea et al. [109] studied the AcoD of CG and cattle manure in a pilot-scale Induced Bed Reactor (IBR) plant with a volume equal to 1250 L.

**Figure 5:**
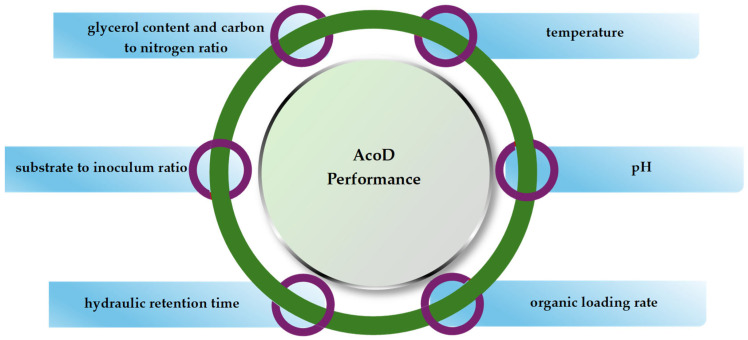
The factors affecting the performance of anaerobic co-digestion process.

As can be seen in Figure 5, among the most commonly reported factors influencing the AcoD performance with the use of CG performance are glycerol content, C/N ratio, temperature, pH, substrate-to-inoculum (S/I) ratio, as well as organic loading rate (OLR) and hydraulic retention time (HRT).

In the present study, the analysis has been performed for the following parameters: CG content and C/N (Section 3.1), as well as temperature and pH (Section 3.2).

**Table 3:** Performance of anaerobic co-digestion process with the use of crude glycerol as a co-substrate: literature data.

Feedstock	Scale	S/I Ratio	Glycerol Content [%*v*/*v*] or [g/L]	C/N	AcoD Conditions				AcoD Performance with the CG Addition					AD Performance Without CG	Ref.
					T [°C]	pH	HRT [d] or [h]	OLR [kgCOD_red_/m^3^/d] or [kgVS_red_/m^3^/d] or [(gVS_red_/L)]	Biogas [mL/gVS_red_] or [mL/gCOD_red_] or (L/L/d)	Methane [mLCH_4_/gVS] or [mLCH_4_/gCOD_red_] or [(mL/d)]	Biogas [mL/gVS_red_/d] or [mL/gCOD_red_/d] or [(L/d)]	Methane [mLCH_4_/gVS_red_/d] or [molCH_4_/m^3^/d] or [(L/L/d)]	CH_4_ [%]		
CG + animal manure	laboratory	-	13.8	-	35	-	-	-	-	-	-	79	-	Methane: 36 mL/gVS/d	[62]
CG + animal manure	laboratory	-	27.5	-	35	-	-	-	-	-	-	57	-		[62]
CG + animal manure	laboratory	-	55.0	-	35	-	-	-	-	-	-	3	-		[62]
CG + animal manure	laboratory	-	110.0	-	35	-	-	-	-	-	-	0	-		[62]
CG + animal manure	laboratory	-	5	-	25.4–28.8	initial: 8.2, final: 5.4	-	-	235	-	-	-	14.9	Biogas: 230 mL/gVS	[65]
CG + animal manure	laboratory	-	10	-	25.4–28.8	initial: 8.8, final: 5.4	-	-	380	-	-	-	3.5		[65]
CG + animal manure	laboratory	-	15	-	25.4–28.8	initial: 9.2, final: 6.4	-	-	0	0	0	0	0		[65]
CG + animal manure	laboratory	1.1	6	29.08	-	initial: 8.18 ± 0.18, final: 7.38 ± 0.12	-	-	-	340.01 ± 5.52	-	-	52.04–72.16	-	[72]
CG + animal manure	laboratory	1.7	6	17.82	-	initial: 8.13 ± 0.19, final: 7.29 ± 0.19	-	-	-	330.33 ± 8.05	-	-	61.90		[72]
CG + animal manure	laboratory	2.3	15	53.89	-	initial: 8.56 ± 0.15, final: 6.33 ± 0.11	-	-	-	172.75 ± 4.82	-	-	59.31		[72]
CG + animal manure	laboratory	2.9	15	26.28	-	initial: 8.52 ± 0.11, final: 7.50	-	-	-	344.13 ± 12.31	-	-	55.43		[72]
CG + animal manure	laboratory	1.1	3.75	17.47	-	initial: 7.95 ± 0.23, final: 7.26 ± 0.03	-	-	-	328.62 ± 8.56	-	-	52.04		[72]
CG + animal manure	laboratory	2.8	16.86	35.85	-	initial: 8.60 ± 0.04, final: 7.00 ± 0.19	-	-	-	199.48 ± 8.69	-	-	61.33		[72]
CG + animal manure	laboratory	1.5	10.5	61.82	-	initial: 8.43 ± 0.06, final: 7.52 ± 0.14	-	-	-	287.13 ± 6.45	-	-	60.68		[72]
CG + animal manure	laboratory	2.4	10.5	20.86	-	initial: 8.31 ± 0.03, final: 7.45 ± 0.05	-	-	-	264.38 ± 12.45	-	-	71.94		[72]
CG + animal manure	laboratory	2.0	10.5	26.98	-	initial: 8.27 ± 0.03, final: 7.56	-	-	-	226.75 ± 16.38	-	-	69.95		[72]
CG + animal manure	laboratory	-	3	27	55	7.7 ± 0.1	-	2.6 ± 0.1	470	-	-	-	-	Biogas: 170 ± 0.01 mL/gVS	[78]
CG + animal manure	laboratory	-	2	-	34 ± 1.0	7.61 ± 0.15	30	2.2	-	336 ± 53	(28.6 ± 1.5)	-	71.3 ± 1.5	-	[83]
CG + animal manure	laboratory	-	4	-	34 ± 1.0	7.72 ± 0.08	30	2.5	-	340 ± 62	(30.8 ± 1.4)	-	68.2 ± 3.0		[83]
CG + animal manure	laboratory	-	6	-	34 ± 1.0	7.85 ± 0.09	30	2.9	-	423 ± 41	(43.6 ± 2.7)	-	69.8 ± 1.9		[83]
CG + animal manure	laboratory	-	8	-	34 ± 1.0	7.85 ± 0.10	30	3.7	-	380 ± 59	(51.1 ± 7.1)	-	68.7 ± 0.8		[83]
CG + animal manure	laboratory	3:4	20	-	35	-	-	-	-	249.6	-	-	-	Methane: 187.9 mLCH_4_/gVS	[89]
CG + animal manure	laboratory	3:4	40	-	35	-	-	-	-	134.1	-	-	-		[89]
CG + animal manure	laboratory	3:4	60	-	35	-	-	-	-	92.9	-	-	-		[89]
CG + animal manure	laboratory	3:4	80	-	35	-	-	-	-	73.3	-	-	-		[89]
CG + animal manure	laboratory	-	2	-	34 ± 1	8.5 ± 0.1	30	- ^a^	-	100 ± 20	(2.13 ± 0.2)	-	62.6 ± 2.4	-	[90]
CG + animal manure	laboratory	-	5	-	34 ± 1	8.4 ± 0.1	30	- ^b^	-	140 ± 30	(3.84 ± 0.3)	-	62.4 ± 2.1		[90]
CG + animal manure	laboratory	-	8	-	34 ± 1	8.3 ± 0.2	30	- ^c^	-	170 ± 30	(5.37 ± 0.3)	-	62.4 ± 2.1		[90]
CG + animal manure	laboratory	0.86–2.27	-	17.88–63.63	30 ± 1.0	7.2–7.6	-	-	458.38–834.57; 278.19–521.46	-	8.64–14.75	-	-	-	[91]
CG + animal manure	laboratory	1:2	4	-	34 ± 1	-	30	-	-	349.0 ± 27.0	-	-	-	Methane: 202.0 ± 14.2 mLCH_4_/gVS	[92]
CG + animal manure	laboratory	1:2	8	-	34 ± 1	-	30	-	-	413.2 ± 28.8	-	-	-		[92]
CG + animal manure	laboratory	1:2	12	-	34 ± 1	-	30	-	-	408.6 ± 25.3	-	-	-		[92]
CG + animal manure	laboratory	1:2	16	-	34 ± 1	-	30	-	-	467.5 ± 26.0	-	-	-		[92]
CG + animal manure	laboratory	-	1	-	37	8.3 ± 0.1	17	1.17 ± 0.07	(0.81 ± 0.06)	480 ± 40	-	-	-	Biogas: 0.46 ± 0.02 L/L/d; 0.41 ± 0.02 L/L/d Methane: 330 ± 80 mL/gVS; 350 ± 70 mL/gVS	[93]
CG + animal manure	laboratory	-	1	-	37	8.3 ± 0.1	22	0.91 ± 0.09	(0.60 ± 0.04)	470 ± 40	-	-	-		[93]
CG + animal manure	laboratory	-	3	-	37	8.2	17	1.91 ± 0.13	(1.48 ± 0.13)	480 ± 40	-	-	-		[93]
CG + animal manure	laboratory	-	3	-	37	8.2 ± 0.1	22	1.42 ± 0.08	(1.01 ± 0.07)	470 ± 20	-	-	-		[93]
CG + sewage sludge	laboratory	-	-	-	37	7.29 ± 0.25	32	-	-	-	920	-	-	Biogas: 350 mL/gVS/d	[17]
CG + sewage sludge	laboratory	1:2	1	14.1	-	initial: 7.55, final: 7.16	-	-	269.2	223.8	30.6	21.4	-	Biogas: 161 mL/gVS, Methane: 138.2 mL/gVS	[40]
CG + sewage sludge	laboratory	1:2	3	17.3	-	initial: 7.48, final: 7.02	-	-	484.7	368.8	56.8	-	-		[40]
CG + sewage sludge	laboratory	-	1	-	35	6.8-7.4	-	-	-	(2353 ± 94)	(1.253 ± 0.163)	-	-	-	[53]
CG + sewage sludge	laboratory	-	0.5	-	37	-	17	0.4	-	-	-	(0.799 ± 0.069)	-	Methane: 0.522 ± 0.048 L/L/d	[54]
CG + sewage sludge	laboratory	-	0.5	-	37	-	17	0.4	-	-	-	(0.941 ± 0.112)	-		[54]
CG + sewage sludge	laboratory	-	2	-	37	-	17	1.5	-	-	-	(1.248 ± 0.058)	-		[54]
CG + sewage sludge	laboratory	-	0.5	-	37	-	17	0.4	-	-	-	(0.780 ± 0.053)	-		[54]
CG + sewage sludge	pilot	-	-	-	35	-	-	1.2	-	358	-	-	-	-	[57]
CG + sewage sludge	pilot	-	3	-	35.0 ± 0.1	-	20	-	- ^e^	-	-	-	59.4; 59.7		[57]
CG + sewage sludge	laboratory	-	10	-	38	initial: 7.90, final: 7.93	56	-	-	428 ± 1	-	-	57 ^g^	Methane: 85 ± 1 mL/gVS	[77]
CG + sewage sludge	laboratory	-	5	-	-	initial: 7.3, final: 4.9	-	-	-	0	-	-	0.4	Methane: 79.0 ± 0.5%	[85]
CG + sewage sludge	laboratory	-	10	-	-	initial: 7.2, final: 6.7	-	-	-	0	-	-	0.2		[85]
CG + sewage sludge	laboratory	-	15	-	-	initial: 7.2, final: 5.5	-	-	-	0	-	-	0.2		[85]
CG + sewage sludge	laboratory	-	20	-	-	initial: 7.3, final: 5.2	-	-	-	0	-	-	0.1		[85]
CG + sewage sludge	pilot	-	2	-	37	6.8–7.2	12.3	1.0–1.7	-	-	(114 ± 1.7)	-	-	Biogas: 30 ± 2.1 L/d	[86]
CG + sewage sludge	pilot	-	2	-	37	6.8–7.2	14.0	1.0–1.7	-	-	(100 ± 8.0)	-	-		[86]
CG + sewage sludge	pilot	-	2	-	37	6.8–7.2	16.4	1.0–1.7	-	-	(90 ± 2.8)	-	-		[86]
CG + sewage sludge	pilot	-	2	-	37	6.8–7.2	19.7	1.0–1.7	-	-	(80 ± 2.6)	-	-		[86]
CG + sewage sludge	pilot	-	2	-	37	6.8–7.2	12.3	1.0–1.7	-	-	(139 ± 7.4)	-	-		[86]
CG + sewage sludge	pilot	-	2	-	37	6.8–7.2	14.0	1.0–1.7	-	-	(130 ± 5.0)	-	-		[86]
CG + sewage sludge	pilot	-	2	-	37	6.8–7.2	16.4	1.0–1.7	-	-	(105 ± 5.5)	-	-		[86]
CG + sewage sludge	pilot	-	3	-	37	6.8–7.2	19.7	1.0–1.7	-	-	(86.5 ± 3.8)	-	-		[86]
CG + sewage sludge	pilot	-	4	-	37	6.8–7.2	19.7	1.0–1.7	-	-	0	-	-		[86]
CG + sewage sludge	laboratory	-	1	-	35	-	5–20	1.03–4.05	-	-	-	(0.6–0.9)	-	-	[107]
CG + cattle manure	laboratory	-	5	-	-	initial: 8.0, final: 7.8	-	-	360 mLCH_4_/gVS_ad_	270 mLCH_4_/gVS_ad_	-	-	74	Biogas: 250 mL/gVS_ad_, Methane: 240 mL/gVS_ad_, Methane: 68.1%	[76]
CG + cattle manure	laboratory	-	10	-	-	initial: 8.0, final: 7.7	-	-	350 mLCH_4_/gVS_ad_	260 mLCH_4_/gVS_ad_	-	-	73		[76]
CG + cattle manure	laboratory	-	15	-	-	initial: 8.0, final: 7.6	-	-	330 mLCH_4_/gVS_ad_	210 mLCH_4_/gVS_ad_	-	-	71		[76]
CG + cattle manure	laboratory	-	20	-	-	initial: 8.0, final: 7.3	-	-	230 mLCH_4_/gVS_ad_	160 mLCH_4_/gVS_ad_	-	-	67		[76]
CG + cattle manure	laboratory	-	6	-	55 ± 0.1	initial: 7.2 ± 0.1, final: 6.7 ± 0.3	18	6.01; 3.24	-	600	-	-	67	-	[82]
CG + cattle manure	laboratory	-	6	-	55 ± 0.1	initial: 7.2 ± 0.1, final: 6.7 ± 0.5	20	5.41; 2.91	-	0	-	-	0		[82]
CG + cattle manure	laboratory	-	6	-	55 ± 0.1	initial: 7.1 ± 0.2, final: 6.8 ± 0.1	22	4.65; 2.35	-	0	-	-	0		[82]
CG + cattle manure	laboratory	-	-	-	39	7.53	30	2.3	-	0.4 ^g^	-	(0.8) ^g^	69	-	[87]
CG + cattle manure	pilot	-	6	28.0 ± 0.50	55 ± 1	initial: 7.82 ± 0.01, final: 7.35 ± 0.07	20	5.8	-	-	-	-	64.48 ± 0.17	-	[109]
CG + cattle manure	laboratory	-	5	-	35–37	-	-	-	825.3	-	-	-	9.5	Biogas: 268.6 mL/gVS	[139]
CG + cattle manure	laboratory	-	10	-	35–37	-	-	-	825.7	-	-	-	14.3		[139]
CG + cattle manure	laboratory	-	15	-	35–37	-	-	-	387.9	-	-	-	14.6		[139]
CG + leachate	laboratory	-	5	-	30 ± 1	final: 8.02 ± 0.28	-	2	-	110 ± 60; (360 ± 170)	-	(0.12 ± 0.35)	-	-	[61]
CG + leachate	laboratory	-	5	-	30 ± 1	final: 8.67 ± 0.20	-	3.5	-	180 ± 90; (3290 ± 1500)	-	(0.86 ± 0.43)	-		[61]
CG + leachate	laboratory	-	5	-	30 ± 1	final: 8.01 ± 0.55	35.2	7.1	-	180 ± 76; (4970 ± 1840)	-	(1.51 ± 0.66)	-		[61]
CG + leachate	laboratory	-	5	-	30 ± 1	final: 7.97 ± 0.41	32.3	11.6	-	80 ± 30; (3770 ± 1440)	-	(1.27 ± 0.45)	-		[61]
CG + distillery wastewater	laboratory	-	5	27.02	- ^f^	7.85	20–35	-	-	339	-	-	27.02	Methane: 265 mL/gCOD	[66]
CG + distillery wastewater	laboratory	-	1	25.40	- ^f^	7.73	20–35	-	-	289	-	-	25.40		[66]
CG + distillery wastewater	laboratory	-	2	25.78	- ^f^	7.75	20–35	-	-	277	-	-	25.78		[66]
CG + distillery wastewater	laboratory	-	3	26.18	- ^f^	7.80	20–35	-	-	271	-	-	26.18		[66]
CG + distillery wastewater	laboratory	-	4	25.60	- ^f^	7.81	20–35	-	-	270	-	-	25.60		[66]
CG + food waste	laboratory	-	10	-	38	initial: 8.16, final: 7.94	56	-	882	442 ± 15	-	-	60.5 ^g^	Methane: 316 ± 7 mL/gVS	[77]
CG + milk sludge	laboratory	-	10	-	38	initial: 7.88, final: 8.04	56	-	858	496 ± 12	-	-	61.58	Methane: 263 ± 1 mL/gVS	[77]
CG + milk sludge + food waste	laboratory	-	10	-	38	initial: 7.89, final: 7.40	56	-	-	409 ± 10	-	-	58 ^g^	-	[77]
CG + sewage sludge + food waste	laboratory	-	10	-	38	initial: 8.37, final: 7.32	56	-	-	338 ± 3	-	-	56 ^g^	-	[77]
CG + sewage sludge + food waste	laboratory	1:2	1	18.3	-	initial: 7.48, final: 7.07	-	-	432.4	343.3	55.1	36.2	-	-	[79]
CG + sewage sludge + food waste	laboratory	1:2	3	38.4	-	initial: 7.13, final: 7.05	-	-	692.6	525.7	79.9	-	-		[79]
CG + seafood wastewater	laboratory	-	1–10	-	-	6.9–8.3	13–51	2	2–577	-	-	-	-	CH4: 278 mL/gVS	[88]
CG + agro-industrial waste	pilot	-	2.5	-	38	7.1–7.5	28.9 ± 0.5	-	-	-	(480 ± 230)	-	-	-	[138]
CG + agro-industrial waste	pilot	-	2.5	-	38	7.1–7.5	86.8 ± 0.2	-	-	-	(144 ± 35)	-	-		[138]
CG + agro-industrial waste	pilot	-	2.5	-	38	7.1-7.5	43.4 ± 0.3	-	-	-	(360 ± 103)	-	-		[138]
CG + agro-industrial waste	pilot	-	2.5	-	38	7.1-7.5	33.3 ± 0.4	-	-	681 ± 98	(576 ± 84)	-	60–70		[138]
CG + sardine wastewater	laboratory	1:1	-	27	37	final: 7.28	-	-	-	244.85	-	-	73.15	Methane: 62.15%	[68]
CG + sardine wastewater	laboratory	2:1	-	43	50	final: 7.52	-	-	-	255.21	-	-	68.48	Methane: 37.28%	[68]
CG + sardine wastewater	laboratory	1:1	1	27	37	7	-	-	-	244.85	-	-	73.15	Methane: 68.21 mL/gCOD	[73]
CG + sardine wastewater	laboratory	1:1	2	43	37	7	-	-	-	78.42	-	-	60.81		[73]
CG + sardine wastewater	laboratory	1:1	3	51	37	7	-	-	-	67.45	-	-	64.12		[73]
CG + sardine wastewater	laboratory	1:1	4	63	37	7	-	-	-	27.38	-	-	63.71		[73]
CG + sardine wastewater	laboratory	1:1	5	73	37	7	-	-	-	21.92	-	-	60.25		[73]
CG + sardine wastewater	laboratory	1:1	1	27	50	7	-	-	-	210.5	-	-	70.57	Methane: 16.29 mL/gCOD	[73]
CG + sardine wastewater	laboratory	1:1	2	43	50	7	-	-	-	255.21	-	-	68.48		[73]
CG + sardine wastewater	laboratory	1:1	3	51	50	7	-	-	-	36.79	-	-	51.40		[73]
CG + sardine wastewater	laboratory	1:1	4	63	50	7	-	-	-	18.03	-	-	33.78		[73]
CG + sardine wastewater	laboratory	1:1	5	73	50	7	-	-	-	10.28	-	-	26.39		[73]
CG + meat and bone meal	laboratory	1:1	-	-	38	initial: 7.57, final: 7.80	-	(27)	-	320	-	-	66.39	-	[59]
CG + meat and bone meal	laboratory	1:1	-	-	38	initial: 7.60, final: 7.79	-	(27)	-	350	-	-	66.33		[59]
CG + meat and bone meal	laboratory	1:1	-	-	38	initial: 7.64, final: 7.77	-	(27)	-	400	-	-	58.67		[59]
CG + dairy wastewater	laboratory	-	2	-	- ^f^	7.21 ± 0.05	-	-	722.0 ± 20.6	-	-	-	74.99 ± 13.52	Biogas: 414.9 ± 57.0 mL/gVS, Methane: 70.40 ± 17.06%	[81]
CG + dairy wastewater	laboratory	-	4	-	- ^f^	7.13 ± 0.04	-	-	1310.0 ± 144.4	-	-	-	73.97 ± 18.44		[81]
CG + dairy wastewater	laboratory	-	8	-	- ^f^	7.07 ± 0.03	-	-	2307.2 ± 312.8	-	-	-	73.10 ± 24.03		[81]
CG + dairy wastewater + meat and bone meal	laboratory	-	13	13	38	initial: 6.77, final: 7.67	30	2.65	1390 ± 10	900 ± 10	-	-	-	-	[75]
CG + municipal wastewater sludge	pilot	-	-	-	36 ± 1	final: 7.25 ± 0.07	-	2.34 ± 0.08; 1.03 ± 0.01	(1.45)	-	-	(0.93)	64.00 ± 1.17	Methane: 60.00 ± 0.84%; 60.00 ± 0.74%; 60.00 ± 0.63%	[3]
CG + municipal wastewater sludge	pilot	-	1.1	-	36 ± 1	final: 7.18 ± 0.05	-	2.38 ± 0.06; 1.04 ± 0.04	-	-	-	-	66.50 ± 2.02		[3]
CG + municipal wastewater sludge	pilot	-	1.8	-	36 ± 1	final: 7.09 ± 0.05	-	2.88 ± 0.11; 1.18 ± 0.04	-	-	-	-	56 ± 1.68		[3]
CG + municipal wastewater sludge	laboratory	-	1.25	-	37	7.45 ± 0.03	-	4.82	-	-	(12.2 ± 0.2)	-	-	Biogas: 8.2 ± 0.1 L/d; 4.5 ± 0.1 L/d	[80]
CG + municipal wastewater sludge	laboratory	-	1.35	-	37	7.30 ± 0.03	-	3.02	-	-	(9.0 ± 0.1)	-	-		[80]
CG + municipal wastewater sludge	laboratory	-	2.72	-	37	6.20 ± 0.10	-	4.01	-	-	(4.8 ± 0.1)	-	-		[80]
CG + municipal solid waste	laboratory	1:1	-	-	35	6.8-7.0	-	-	-	(2094 ± 92)	-	-	-	Metane: 1400 ± 305 mL/d	[58]
CG + palm oil mill final	laboratory	-	1	-	37	initial: 6.89, final: 7.35	-	-	-	553.46, 276.73, (45.66)	-	-	-	Methane: 278.64 mL/gVS; 73.34 mL/d	[74]
CG + palm oil mill final	laboratory	-	2	-	37	initial: 6.92, final: 5.34	-	-	-	98.24, 49.12, (26.51)	-	-	-		[74]
CG + palm oil mill final	laboratory	-	3	-	37	initial: 6.99, final: 5.35	-	-	-	77.48, 38.74, (11.97)	-	-	-		[74]
CG + palm oil mill final	laboratory	-	4	-	37	initial: 7.08, final: 5.30	-	-	-	62.36, 31.18, (10.09)	-	-	-		[74]
CG + palm oil mill final	laboratory	-	5	-	37	initial: 7.15, final: 5.30	-	-	-	55.47, 27.87, (8.95)	-	-	-		[74]
CG + sugarcane stillage	laboratory	-	1.53	-	30	initial: 7.9 ± 0.2, final: 7.7 ± 0.2	-	5	-	-	-	60.23	81.9 ± 0.2	Methane: 83.2 ± 0.5%	[140]
CG + sugarcane stillage	laboratory	-	1.53	-	30	initial: 7.55 ± 0.04, final: 7.5 ± 0.1	-	5	-	-	-	59.61	83 ± 1		[140]
CG + sugarcane stillage	laboratory	-	1.53	-	30	initial: 7.4 ± 0.3, final: 7.7 ± 0.2	-	5	-	-	-	58.57	83.5 ± 0.3		[140]
CG + sugarcane stillage	laboratory	-	1.53	-	30	initial: 7.6 ± 0.1, final: 8.2 ± 0.3	-	5	-	-	-	65.39	80.5 ± 0.1		[140]
CG + sugarcane stillage	laboratory	-	1.53	-	30	initial: 7.40 ± 0.09, final: 7.3 ± 0.2	-	5	-	-	-	68.57	84.0 ± 0.6		[140]
CG + sugarcane stillage	laboratory	-	1.53	-	30	initial: 7.5 ± 0.1, final: 7.4 ± 0.3	-	5	-	-	-	52.10	86.0 ± 0.4		[140]
CG + sugarcane stillage	laboratory	-	1.53	-	30	initial: 7.5 ± 0.1, final: 7.6 ± 0.1	-	7.5	-	-	-	95.07	82.6 ± 0.2		[140]
CG + sugarcane stillage	laboratory	-	1.53	-	30	initial: 7.4 ± 0.1, final: 7.7 ± 0.3	-	10	-	-	-	139.32	83.1 ± 0.1		[140]
CG + sugarcane stillage	laboratory	-	1.53	-	35	initial: 7.5 ± 0.2, final: 8.2 ± 0.2	-	10	-	-	-	122.99	83.2 ± 0.7		[140]
CG + olive mill wastewater + slaughterhouse wastewater	laboratory	1:1	-	-	35	6.9–7.6	-	-	-	(1210 ± 205)	-	-	-	Methane: 479 mL/d	[58]

CG—crude glycerol; S/I—substrate to inoculum ratio; C/N—carbon to nitrogen ratio; T—temperature; HRT—hydraulic retention time; OLR—organic loading rate; ^a^ 13.0 ± 0.4 g/d; ^b^ 17.5 ± 0.6 g/d; ^c^ 19.5 ± 0.5 g/d; ^e^ 0.65 m^3^/L of glycerol; ^f^ mesophilic conditions; ^g^ data from the graph.

### 3.1. Crude Glycerol Content and C/N Ratio

The anaerobic digestion of organic waste is a complex biological process that involves a series of metabolic methods, such as hydrolysis, acidogenesis, acetogenesis and methanogenesis. Mono-digestion is limited by several factors, for instance [141,142]:

(i) Low methane production of methane;
(ii) Long retention time of digestion process;
(iii) Low efficiency of volatile solids reduction.

By contrast, the use of CG as a co-substrate for the biogas production via AcoD provides a great improvement of the process yield. This finding has been widely confirmed in the literature for results obtained for AcoD conducted under both mesophilic and thermophilic conditions with the use of various substrates.

With regard to digestion of sewage sludge under mesophilic conditions, Alves et al. [79] demonstrated that the addition of raw glycerol led to the increase in the biogas and methane yield by 67% and 62%, respectively. These results are in agreement with those obtained by Fountoulaki et al. [53], who found that the addition of CG enhanced CH_4_ production by about 1247 mL/d. In turn, in [67] it has been documented that this type of co-digestion improved biogas production by 4.5 times.

Likewise, in [87] it has been noted that the addition of glycerine phase to cattle manure allowed the generation of 3.1 times more biogas and a 10% higher CH_4_ content compared to the those obtained for the AD of only cattle manure. In another study [89], it has been indicated that the mixture of crude glycerol and animal manure provided higher methane production by 125% compared to that noted for mono-digestion. Other authors [93] concluded that supplementation of feedstock with CG led to a 222% increase in biogas productivity compared to manure mono-digestion. Furthermore, it has been documented that adding raw glycerol to distillery wastewater allowed the improvement of the methane yield by 29%. It should be pointed out that similar observations have been noted for the process performed with the use of three substrates. Indeed, in [58] it has been found that the addition of raw glycerol to olive mill wastewater and slaughterhouse wastewater allowed an increase in the CH_4_ production by about 731 mL/d.

It is necessary to mention that with regards to AcoD performed under thermophilic conditions, in [78] it has been demonstrated that the addition of glycerol resulted in a higher specific biogas production by 0.30 L/gVS compared to that obtained for the mono-digestion of animal manure. In addition, Srimachai et al. [68] have demonstrated that the use of CG as a co-substrate for thermophilic AcoD led to the increase in the methane production by 40.76 compared to the mono-digestion of canned sardine wastewater.

Performing the literature review allowed us to show that CG was used as a co-substrate for AcoD in a wide range of concentrations (Table 3). Indeed, its content in the feedstocks was in the range from 0.5% *v*/*v* [54] to 20% *v*/*v* [85]. It is important to point out that the CG content may have a significant impact on the AcoD process performance. This phenomenon has been investigated in several papers wherein experiments with the use of two [40,62,65,73,81,83,90,93] and three [79] substrates have been conducted. Alves et al. [40] have investigated the impact of CG concentration on the AcoD performance under mesophilic conditions with the use of sewage sludge (Table 2) as a substrate. The above-mentioned authors have noted that higher CG concentration provided higher values of performance. For feedstock containing 1% *v*/*v* (C/N of 14.1) and 3% *v*/*v* (C/N of 17.3) of CG, the methane yield was equal to 0.2338 mLCH_4_/gVS and 0.3688 mLCH_4_/gVS, respectively (Table 3). This observation is in accordance with the relative findings reported in [93], wherein AcoD of CG and animal manure (Table 2) was studied. It has been reported that CG content has a great impact on the process. More specifically, it has been recognized that under HRT of 17 and 22 days, the increase in the CG concentration from 1% *v*/*v* to 3% *v*/*v* led to an improvement in the biogas productivity from 0.81 ± 0.06 L/L/day to 1.48 ± 0.13 L/L/day and from 0.60 ± 0.04 L/L/day to 1.01 ± 0.07 L/L/day, respectively (Table 3). A higher range of CG content co-digested with animal manure (Table 2) has been applied in [90], wherein it has been found that for CG concentration of 2% *v*/*v*, 5% *v*/*v* and 8% *v*/*v,* biogas production was 2.13 ± 0.2 L/day, 3.84 ± 0.3 L/day and 5.37 ± 0.3 L/day, respectively (Table 3). It is important to note that the increase in the amount of CG ensured high quality of the biogas produced. Indeed, for all applied CG concentrations, the CH_4_ content in biogas was equal to around 63%. A similar finding has been presented for AcoD of feedstock containing three substrates: CG, food waste and sewage sludge (Table 2) [79]. Indeed, it has been documented that increasing CG content from 1% *v*/*v* to 3% *v*/*v* allowed an enhancement of the methane and biogas yield from 343.3 mLCH_4_/gVS to 525.7 mLCH_4_/gVS and from 432.4 mL/gVS to 692.6 mL/gVS, respectively (Table 3). Therefore, it can be indicated that with a few exceptions, the increase in the CG content led to an increase in the biogas yield.

Although several studies focusing on the impact of CG on the AcoD performance have been performed, there are still some significant issues related to this correlation. It is important to note that adding CG to the system may lead to an inhibitory effect. Indeed, this phenomenon has been reported in the literature for co-digestion of CG with various substrates such as animal manure [72,83,92], sewage sludge [86], cattle manure [76,139] and municipal wastewater sludge [80]. For instance, in [76] it has been found that for AcoD of CG and cattle manure (Table 2), a concentration of CG greater than 6% led to a decrease in the process performance (Table 3). Indeed, in the above-mentioned paper, it has been indicated that this phenomenon could be a result of overload of organic material in digestion, which in turn was associated with the low quality of the CG. More precisely, the used CG was characterized by a high content of lipids (78%), which may have a negative effect on methanogenic archaea in the case of high loading rates. These findings are similar to those reported by Athanasoulia et al. [86], who indicated that adding 4% of CG to sewage sludge (Table 2) led to the failure of the AcoD system due to overloading (Table 3). In [92] the failure of the AcoD process of animal manure (Table 2) and CG was noted for CG content equal to 8% (Table 3). It has been explained by the high evolution of H_2_S in produced biogas and accumulation of VFA. In turn, Razaviarani et al. [80] demonstrated that adding CG to municipal wastewater sludge at the content of 2.72% *v*/*v* led to the significant decrease in the biogas production and methane yield (Table 3). Likewise, in [139] it has been found that increasing the CG concentration used for AcoD with cattle slurry led to a decrease in the process performance. The biogas yield for the CG concentration of 5%, 10% and 15% was equal to 825.3 mL/gVS_red_, 825.7 mL/gVS_red_ and 387.9 mL/gVS_red_, respectively (Table 3). The authors have pointed out that this observation could be caused by the inhibition of the AcoD process due to higher concentrations of methanol and KOH present in the substrate resulting from the increase in glycerol content.

**Figure 6:**
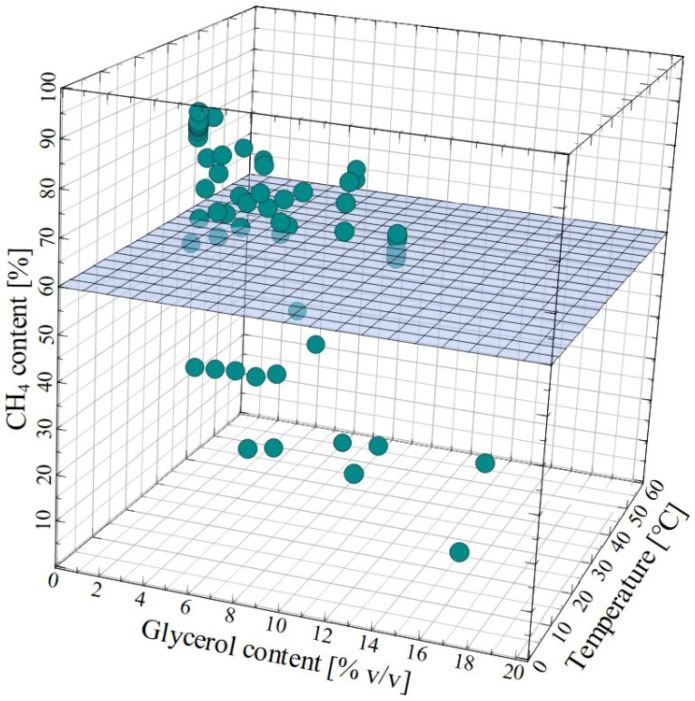
The impact of crude glycerol content on the methane content in biogas produced during the AcoD of crude glycerol mixed with various substrates performed under mesophilic and thermophilic conditions. Literature data.

It should therefore be clearly emphasized that in the case of AcoD using CG as co-substrate, its suitable concentration should be carefully analyzed. Figure 6 demonstrates the impact of CG content in the feed on the CH_4_ content in the biogas produced during the AcoD of CG mixed with various substrates performed under mesophilic and thermophilic conditions, based on the data presented in the literature. It has been found that for most of the experiments performed, CH_4_ content in the biogas higher than 60% has been obtained under CG content up to 8% *v*/*v*. This finding may be key in determining the most suitable concentration of raw glycerol used for biogas production via AcoD.

At the same time, it should be noted that generally, the addition of CG leads to an increase in the C/N ratio. During anaerobic digestion, carbon is used as the energy source, while nitrogen is a source of nutrition used by microorganisms to form the body cells. Hence, it is one of the key factors determining the AcoD performance. In short, with regards to AD, in has been indicated that the optimum C/N value is between 16:1 and 33:1 [96]. Nevertheless, according to [143], determining the optimum value of C/N ratio for AcoD is a great challenge since it can be influenced by several parameters, including:

(i) Type of substrate;
(ii) Content of trace elements;
(iii) Chemical components present in the feedstock;
(iv) Biodegradability.

The analysis performed in the current study has shown that AcoD with CG used as a substrate has been performed under a wide range of C/N (Table 3). Indeed, C/N was applied in the range from 14.1 [40] to 73 [73]. It is worth noting that, in [91], it has been shown that it may have a significant impact on the abundance of the bacterial classes involved in the feedstock treatment and thus, on the AcoD performance (Table 3). However, in order to determine the most suitable range of C/N values, more studies are required.

### 3.2. Temperature and pH

As mentioned previously, temperature is one of the most important factors influencing the AD performance. Overall, the process can be operated under the following temperature regimes: psychrophilic (10–20 °C), mesophilic (30–40 °C) and thermophilic (50–60 °C). Despite a growing number of studies, little is known about the performance of thermophilic AcoD. Indeed, in the present study, it has been found that most of the studies focusing on the AcoD of CG and various substrates have been performed under mesophilic conditions (Table 3). Indeed, thermophilic conditions have been applied only in a few investigations [68,73,82,109]. This can probably be explained by the fact that thermophilic anaerobic digestion is more energy-intensive and is characterized by lower stability [144]. However, it is important to note that in general, thermophilic digestion has several advantages. For instance, it has the potential to ensure higher CH_4_ yield since thermophilic microorganisms are characterized by a higher growth rate and thus provide a higher reaction rate [145]. This finding has been confirmed by results obtained by Srimachai et al. [68], who investigated the AcoD of raw glycerol mixed with sardine wastewater (Table 2) under mesophilic and thermophilic conditions. The above-mentioned authors noted higher methane yield performed at a temperature equal to 55 °C (255.21 mLCH_4_/gCOD_red_) compared to that obtained at 35 °C (244.85 mL CH_4_/gCOD_red_) (Table 3). What becomes apparent from the discussed studies is that an increase in the process temperature above 50 °C may significantly improve the AcoD performance.

It is widely assumed that another important factor when considering the performance of AcoD is the pH feedstock. Obviously, pH affects the microorganisms’ growth and activity. It is worth mentioning that the analysis performed in the present study revealed that pH values of feedstock used for AcoD were in the range from 6.20 ± 0.10 [80] to 8.5 [90] (Table 3). It is in line with the optimum pH value (from 6.8 to 8.0) widely reported in the literature [146,147,148,149]. Finally, it should be pointed out that co-digestion of CG with selected substrates may decrease the cost of chemicals used for pH adjustment during the process. As has been demonstrated by Phuket et al. [66], the use of CG as a co-substrates for AcoD of distillery wastewater (Table 2) led to the increase in the initial pH. Indeed, it was in the range between 7.73 and 7.85 (Table 3), while during mono-digestion it was equal to 3.52.

## 4. Conclusions and Perspectives

In recent years, the global size of the CG market has been growing, primarily due to increasing demand for biodiesel. For this reason, appropriate management of this by-product is required. One of the most interesting applications of raw glycerol is its use for biogas production via the AcoD process.

To the best of the authors’ knowledge, the current study is the first one to demonstrate an analysis of the reported characteristics of crude glycerol and substrates used for AcoD as well as the impact of selected factors on the process performance. Detailed analysis allowed us to show that:

(i) Values of key parameters characterizing the CG (glycerol concentration, pH, TS and VS content as well as COD) used for the AcoD were within wide ranges. It can be attributed to the fact that the CG composition depends mainly on the source of oil used for the biodiesel production, processing technology, ratio of reactants, type of catalyst and procedure applied.
(ii) Adding CG to the feedstock caused a significant enhancement of CH_4_ and biogas yield compared to results obtained for the mono-digestion process of various substrates. Indeed, raw glycerol is a source of additional carbon, which can be consumed by specific microorganisms and converted to CH_4_.
(iii) The increase in the CG concentration in the feedstock leads to an enhancement of the AcoD performance; nevertheless, the substrate inhibition effect should be considered.
(iv) For most of the experiments presented in the literature, CH_4_ content in the biogas higher than 60% has been obtained under CG content up to 8% *v*/*v*. A higher concentration may cause a reduction in the biogas yield due to the overload of organic material in digestion.
(v) Most of studies focusing on the AcoD performance with the use of CG have been carried out by applying laboratory-scale installations; hence, further experimental investigations with the use of pilot-scale systems are recommended.
(vi) In order to evaluate the performance of AcoD with the use of CG under thermophilic conditions, more studies are required.

Finally, this work has highlighted that CG can be successfully used as a co-substrate for biogas production via the AcoD process. Undoubtedly, the present analysis may have important implications for raw glycerol management.

## Data Availability

The data presented in this study are available on request from the corresponding author. The data are not publicly available due to the institutional repository being under construction.

## Abbreviations

AD	Anaerobic digestion
AcoD	Anaerobic co-digestion
C	Carbon
CG	Crude glycerol
COD	Chemical oxygen demand
CSTR	Continuously stirred tank reactor
C/N	Carbon to nitrogen ratio
HRT	Hydraulic retention time
IBR	Induced bed reactor
MW	Molecular weight
N	Nitrogen
OLR	Organic loading rate
S/I	Substrate to inoculum
TN	Total nitrogen
TP	Total phosphorus
TS	Total solids
VS	Volatile solids
